# Neuro-Immune Cross-Talk in the Striatum: From Basal Ganglia Physiology to Circuit Dysfunction

**DOI:** 10.3389/fimmu.2021.644294

**Published:** 2021-04-19

**Authors:** Andrea Mancini, Veronica Ghiglieri, Lucilla Parnetti, Paolo Calabresi, Massimiliano Di Filippo

**Affiliations:** ^1^ Section of Neurology, Department of Medicine and Surgery, Università degli Studi di Perugia, Perugia, Italy; ^2^ Università Telematica San Raffaele, Rome, Italy; ^3^ Section of Neurology, Fondazione Policlinico Universitario Agostino Gemelli IRCCS, Rome, Italy; ^4^ Department of Neuroscience, Università Cattolica del Sacro Cuore, Rome, Italy

**Keywords:** basal ganglia, nucleus striatum, immune system, synaptic transmission, synaptic plasticity

## Abstract

The basal ganglia network is represented by an interconnected group of subcortical nuclei traditionally thought to play a crucial role in motor learning and movement execution. During the last decades, knowledge about basal ganglia physiology significantly evolved and this network is now considered as a key regulator of important cognitive and emotional processes. Accordingly, the disruption of basal ganglia network dynamics represents a crucial pathogenic factor in many neurological and psychiatric disorders. The striatum is the input station of the circuit. Thanks to the synaptic properties of striatal medium spiny neurons (MSNs) and their ability to express synaptic plasticity, the striatum exerts a fundamental integrative and filtering role in the basal ganglia network, influencing the functional output of the whole circuit. Although it is currently established that the immune system is able to regulate neuronal transmission and plasticity in specific cortical areas, the role played by immune molecules and immune/glial cells in the modulation of intra-striatal connections and basal ganglia activity still needs to be clarified. In this manuscript, we review the available evidence of immune-based regulation of synaptic activity in the striatum, also discussing how an abnormal immune activation in this region could be involved in the pathogenesis of inflammatory and degenerative central nervous system (CNS) diseases.

## Highlights

- The basal ganglia network operates for appropriate context-dependent cognitive, behavioral and emotional responses.
- Bidirectional plastic changes of striatal synapses allow input integration and input-output associations in the basal ganglia network.
- Astrocytes gate striatal excitatory synaptic transmission and orchestrate striatal pathways and subnetworks activation.
- Soluble immune molecules may influence striatal glutamatergic transmission acting on both pre- and post-synaptic sites.
- Pathological activation of striatal astrocytes and microglia could influence the synaptic bases of basal ganglia network functioning, leading to cognitive and behavioral abnormalities during neurological disorders.

## Introduction

The extensive research performed during the last years has made it clear the crucial role of the immune system in the field of cognitive and behavioral sciences. Human behavioral, cognitive and social traits could be deeply influenced by the activation of immune cells in both physiological and pathological conditions. The classical concept of the central nervous system (CNS) as an immune-privileged site has significantly evolved during the last years, acknowledging the presence of functional meningeal lymphatic vessels and a complex neuro-immune cross-talk involving innate and adaptive immunity, as well as resident immune cells within the CNS (1, 2). Indeed, the release of soluble immune mediators is thought to physiologically tune the activity of neural networks, influencing learning and memory processes through the regulation of synaptic transmission and long-term plasticity (3–5).

A prototypical example of the neuromodulatory role of the immune system is represented by the shift in an individual’s behavior and perceptions frequently accompanying infectious diseases. Indeed, a reduced interest in social interactions and unnecessary physical activity could represent a protective evolutionistic response aimed at limiting pathogen spreading in a social community. These complex cognitive and behavioral responses are thought to be caused by pro-inflammatory mediators released by immune cells counteracting the infection (6). If the immune activation is inappropriate or unabated, this para-physiological process may become pathological. Indeed, many disabling cognitive and behavioral features occurring during neuroinflammatory and neurodegenerative disorders are thought to rely on the detrimental neuronal and synaptic effects triggered by an uncontrolled cerebral inflammatory microenvironment (4, 7). The influence exerted by the immune system on neuronal and synaptic activity has been mainly investigated in cortical areas, such as in the hippocampus (8, 9), while less is known about the neuro-immune cross-talk occurring in subcortical structures such as the basal ganglia, that together with cortical structures mediate cognitive and behavioral functions (10, 11).

The cortico-striato-thalamo-cortical network was originally described as an essential circuit for locomotor activity and movement execution (12). Still, the identification of extensive functional connections between the striatum and non-motor cortical areas (13, 14) raised the hypothesis of an involvement of the basal ganglia also in associative, cognitive and emotional processes. Indeed, thanks to the closed-loop architecture of the basal ganglia network, the striatum can filter and integrate different cortical inputs during goal-directed behavior, decision making and response selection under competition (11).

The complex microstructural organization of the striatum, characterized by multiple inhibitory and excitatory synaptic connections among various neuronal subtypes, highlights the activity of this structure as an input integrator. Functional or structural alterations of such synaptic connections can powerfully influence the final output and tuning of the whole basal ganglia network (15). Despite the essential functions of the basal ganglia, the potential neuro-immune interactions occurring at this level have been less investigated and should be better understood. In this review, we summarize the available evidence suggesting an immune-based regulation of synaptic activity in the striatum during physiological conditions and pathological inflammatory and degenerative processes of the CNS.

## The Basal Ganglia Network: From Movements to Emotions

The functional anatomy of the basal ganglia can be described as a closed-loop network with two different pathways canonically considered as parallel and opposed, one favoring (direct) and another inhibiting (indirect) the activation of cortical brain areas. The main input of the basal ganglia network is represented by glutamatergic excitatory projections from cortical and thalamic areas, making synaptic contact with striatal medium spiny neurons (MSNs) and aspiny interneurons (16, 17). Such corticostriatal connections are influenced by dopaminergic projections arising from the substantia nigra pars compacta (SNc) and converging into the dendritic tree of MSNs, which can be distinguished by their dopamine (DA) receptor expression patterns (18, 19). In addition, striatal GABAergic or cholinergic interneurons can act as additional elements for the integration of cortical, thalamic, and dopaminergic afferents and the modulation of neighboring MSNs activation (20).

**Figure 1:**
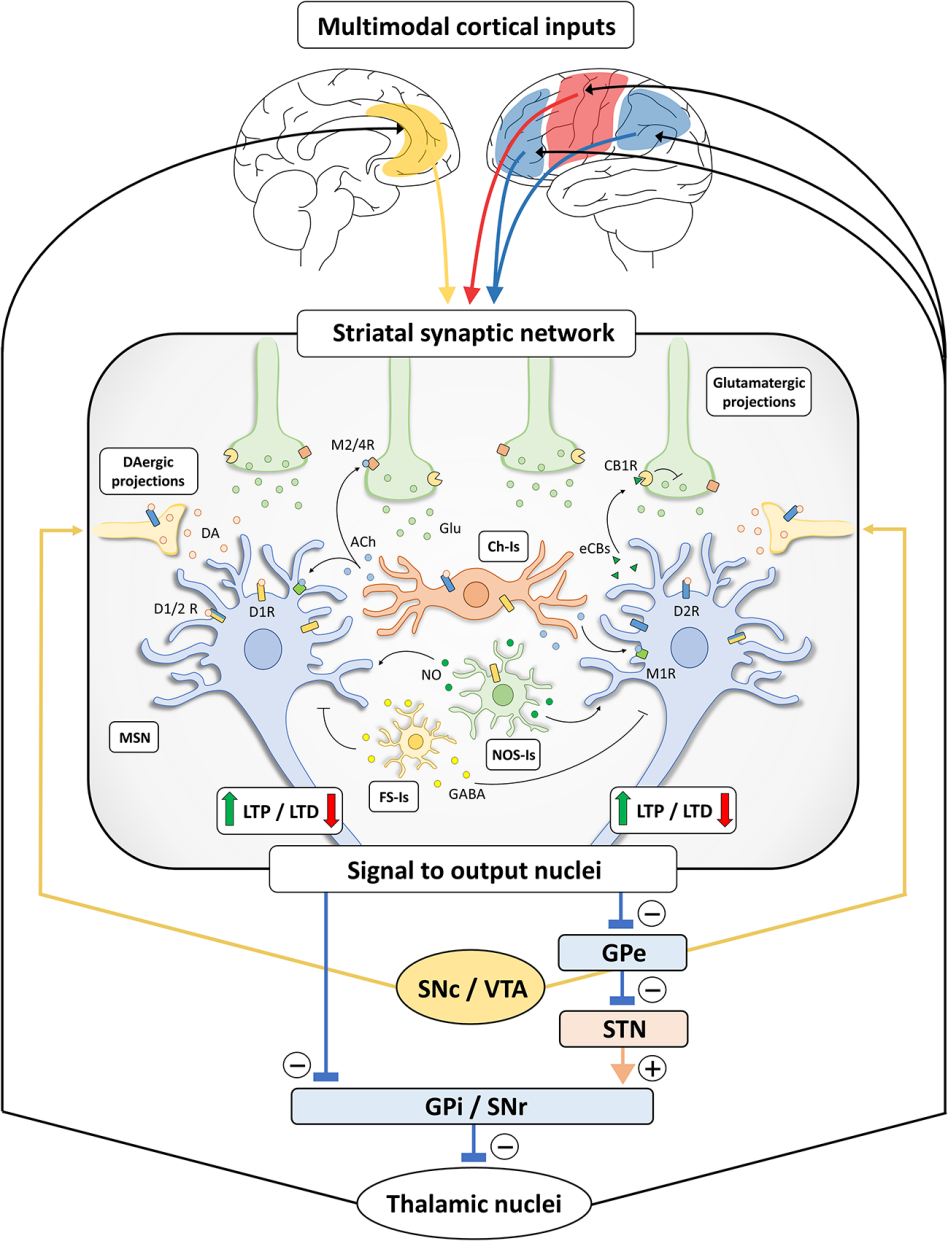
Schematic representation of basal ganglia and striatal synaptic networks. Multimodal inputs are constantly conveyed toward the striatum, including projections arising from sensori-motor cortices (red), limbic structures (yellow) and associative areas (blue) (10, 11, 21). The striatal synaptic network acts as a processing unit through differential signal amplification, output selection and context-dependent input integration. The induction of bidirectional synaptic plastic changes (long-term potentiation, LTP, and long-term depression, LTD) at corticostriatal connections is deeply influenced by DA released by dopaminergic (DAergic) terminals, originating from substantia nigra pars compacta/ventral tegmental area (SNc/VTA). Specifically, LTP of corticostriatal projections is dependent on the activation of D1-like receptors (D1Rs) (22, 23) and under the negative control of D2-like receptors (D2Rs) (24), while the induction of LTD requires the presence of functionally active D1Rs and D2Rs (25–29). These observations are not in line with the classical view of a complete D1- and D2-like receptor functional segregation (30, 31) and may rely on the presence of MSNs expressing both receptor subtypes (32) or membrane heteromeric D1/D2 receptors (D1/2Rs) (33–35). In addition, DA may indirectly act on MSNs through different populations of striatal interneurons (25, 36–38). Striatal cholinergic (Ch-Is), NOS-positive (NOS-Is) and fast-spiking (FS-Is) interneurons exert a feedforward and parallel control of striatal circuit (15). Acetylcholine (Ach) released by Ch-Is can act on M2/4 muscarinic receptors expressed by pre-synaptic glutamatergic terminals and on M1 muscarinic receptors expressed by MSNs. The DA-dependent modulation of Ach release by Ch-Is (expressing both D1Rs and D2Rs) can influence the induction of synaptic LTD in MSNs (39). Nitric oxide (NO) is released by NOS-Is under the control of D1Rs and could act on MSNs facilitating LTD at the post-synaptic level (39). FS-Is releasing GABA represent a parallel inhibitory system. Of note, dopaminergic regulation of LTD induction also relies on the release of retrograde neurotransmitters under the control of different cell-type specific thresholds in D1R- and D2R-expressing MSNs (40). Indeed, the D2R-dependent release of endocannabinoids (eCBs) by MSNs modulates LTD induction through the activation of CB1 cannabinoid receptors (CB1Rs) located on glutamatergic terminals, inhibiting glutamate (Glu) release. Striatal processing of cortical multimodal inputs generated an integrated signal to output nuclei which, in turn, project to thalamic nuclei sending efferents that complete the cortico-basal ganglia-thalamo-cortical loop. Specifically, striatal inhibitory outputs directed toward the GABAergic neurons of substantia nigra pars reticulata (SNr) and globus pallidus pars interna (GPi), which make direct inhibitory synaptic connections with the thalamus, ultimately results in a disinhibition of the thalamic glutamatergic cortical projections (direct pathway). Conversely, the activation of striatal MSNs connected to the globus pallidus pars externa (GPe) results in a disinhibition of the glutamatergic neurons of subthalamic nucleus (STN), leading to a GPi/SNr-dependent inhibition of thalamo-cortical projections (indirect pathway). The presence of bridging collaterals in striatofugal projections ensures signal coordination and mutual inhibition for each pathway and each subnetwork (41, 42). Please, note that the schematic representation of the striatal network does not reflect the effective relative size of the neuronal cells.

The extensive net of intra-striatal inter-neuronal connections, integrating various cortical and sub-cortical inputs, makes the striatum a crucial station from which the information is filtered and channeled through the direct and indirect pathways (**Figure 1**).

The classical “push–pull”, direct–indirect dichotomous view of striatal output pathways has been challenged by the evidence that both pathways are activated during the initiation/stopping of actions or behavioral sequences (11, 43–45) and by the identification of multiple functional and structural connections between the two pathways orchestrating the activity of the whole network (15). The architecture of the basal ganglia network might allow to obtain a simple binary output (go/no-go response) from various cortical and subcortical inputs, perfectly fulfilling their acknowledged role in solving a “selection” problem (46). Accordingly, basal ganglia are considered a phylogenetically conserved network underlying action selection in vertebrates initially devoted to the execution of the previously learned motor plan (47) and subsequently co-opted for other key mammalian superior cortical functions through a process of exaptation, following the evolution of cortical networks (48). Indeed, the striatum receives massive projections from almost all regions of the cortex, acquiring sensorimotor inputs, emotional/motivational information from limbic areas, and multimodal processed data from associative areas (10, 11, 49). This various set of basal ganglia inputs could be considered as a “generator of diversity” (21), from which the striatal filter selects a proper output response which is conveyed through the thalamic nuclei to functionally distinct cortical areas (13, 50). The initially proposed presence of parallel and segregated basal ganglia sub-networks (13), each one processing a different type of input, has been challenged by the description of functional overlap (51–54), allowing the integration of multimodal information in line with the known influence of emotional and motivational state on an individual’s behavior.

Overall, basal ganglia seem to be involved in a wide range of behavioral, cognitive and affective functions, leading to the execution of a specific response out of the different choices continuously arising during daily living. Basal ganglia activity could be involved both in conscious goal-directed behavior and in habitual unconscious actions, representing two possible decision-making performances (21, 55). Ventral and dorsolateral striatal networks seem to be deeply involved in both situations, characterized by the selection of an appropriate action through the evaluation of context-dependent information (21, 55, 56). Extensive afferents from the ventral tegmental area (VTA), the ventral hippocampal subiculum, the prefrontal cortex and the basolateral amygdala converge into the ventral striatum, specifically in the nucleus accumbens (NAc), allowing the integration of contextual/spatial information with affective inputs to select a proper reward-based adaptive action (57–59). In this context, proactive or reactive inhibition of habitual actions is involved not only in motor activity but also in cognitive functions, gating the access to working memory (60, 61), or avoiding the recall of irrelevant information (11) and emotional reactions (like inhibiting context-inappropriate anxious or fear reactions).

According to the critical roles played by the basal ganglia circuit in brain physiology, its dysfunctional activity could lead to a wide range of behavioral/cognitive/emotional consequences. The selection process mediated by the circuit could become altered, since basal ganglia malfunction could be followed by an excessive impulsivity in high-conflict decisions (62), lowering the information threshold required for a selection (63) and influencing the balance between speed and accuracy of performance (64). In line with this view, some clinical characteristics of different neuropsychiatric disorders are thought to rely on an alteration of basal ganglia activity. These include, but are not limited to, bradykinesia, apathy and abulia in Parkinson’s disease (PD); motor or verbal urges in Tourette’s Syndrome (TS); impulsivity and lack of attention in attention-deficit hyperactivity disorder; intrusive thoughts and compulsive behaviors in obsessive-compulsive disorder (OCD); hyperactivity in Huntington’s disease (HD) (11, 65, 66). The pathophysiology of these conditions could rely on the alteration of cellular and synaptic mechanisms underlying the context-dependent selection operated by the basal ganglia network.

## Striatal Synaptic Plasticity and Information Processing in the Basal Ganglia

Since their discovery, long-lasting and activity-dependent plastic changes of synaptic transmission have been considered a plausible biological process underlying brain ability to translate experiences into memories (67–70). Synaptic long-term potentiation (LTP) might enhance the synaptic weight of specific neuronal connections, increasing input specificity of neural network and lasting sufficiently long to induce the formation of stable memories (69, 70). On the other side, long-term depression (LTD) of synaptic connections may enhance input divergences, inhibiting competitive connections or reversing a previous synaptic potentiation due to bidirectional synaptic changes (39, 69, 70).

In this scenario, the synaptic plastic changes described at excitatory corticostriatal connections (15, 39, 71–73) are deeply influenced by the activation of both D1- and D2-like DA receptors (39) and by an extensive net of parallel connections, involving interneurons, such as fast-spiking GABA-releasing cells, large cholinergic neurons, and NO synthase (NOS)-positive interneurons (20, 36, 39, 74, 75) (**Figure 1**).

A fine coordination of striatal direct/indirect MSNs synaptic activity is thought to be crucial for the execution of a specific task (76–80), especially considering that the *in vivo* activation of the two striatal pathways was found to be concurrent (43) and complementary (81, 82) during the execution of motor and behavioral sequences. In addition, learning and refinement of actions seem to require parallel but dissociable input processing within associative and sensorimotor striatal subnetworks, implying a learning-related *in vivo* modulation of corticostriatal synaptic transmission (83) and a dynamic filtering of cortical inputs (84–86).

Behaviorally relevant reinforcement signals might influence striatal synaptic plasticity through short-latency and phasic release of DA from the ascending midbrain projections (87–89). These dopaminergic inputs are thought to play a key role in prediction/learning of reward-related processes by reinforcing causal relationships and input-outcome association during the execution of novel actions (90). This hypothesis is supported by the evidence that appropriately timed dopaminergic reinforcement signals are required to induce corticostriatal bidirectional plasticity, with divergent outcomes depending on the intensity and timing of MSNs activation by cortical/thalamic projections (39, 91). Specifically, it has been shown in intact animals that behaviorally relevant reinforcement signals, inducing a phasic release of DA in the striatum, are required for corticostriatal potentiation, and this occurs only if the electrical stimulation of the motor cortex precedes the depolarization of striatal MSNs (positive paring). Conversely, the same dopaminergic reinforcement is able to induce corticostriatal depression when cortical activation occurs after MSNs membrane depolarization (negative pairing) (91). This form of bidirectional synaptic plasticity, named spike-timing-dependent plasticity (STDP), is considered as a synaptic Hebbian learning paradigm (92, 93) and is deeply influenced by striatal eCBs release, serotonergic transmission, and stimulation of dopaminergic receptors (94–97).

In this scenario, it has been proposed that the input component from the cortex represents an ongoing behavior/action (98), and a positive pairing would arise when a specific cortical projection has directly contributed to MSNs depolarization (91). This striatal synaptic pairing could enhance input specificity and input divergence for a proper behavior/action selection allowing action-outcome association and context-dependent positive selection of satisfactory actions. In parallel, the divergent depressive changes of negatively-paired connections can refine striatal habit formation, lowering the strength of corticostriatal connections not contributing to action yielding reward and increasing signal-to-noise ratio (91).

Of note, the detection of the temporal contingency between two consecutive stimuli requires a balanced removal/reuptake of neurotransmitters previously released in the synaptic cleft. Astroglial cells could be deeply involved in these processes, and an alteration of their homeostatic functions can disrupt the induction of Hebbian synaptic plastic changes, leading to aberrant non-timing-dependent plasticity for uncorrelated events or precluding STDP expression (99).

Overall, the emerging picture of the basal ganglia network organization is more dynamic and fluid than that previously established. Cortical and thalamic inputs can be filtered and integrated in the striatum by the intrinsic membrane plastic properties of MSNs, fluctuating between an “up” or “down” state depending on the firing frequency of cortical inputs (100–102). Input signal specificity and input divergence may be guaranteed and enhanced by bidirectional plastic changes of corticostriatal synapses, under the control of parallel intra-striatal connections among MSNs and interneuronal cells and vertical dopaminergic projections arising from the midbrain, influencing motivational behavior and reward-related learning.

Such a functional view gets away from the simplistic dichotomous model of direct/indirect pathways and focuses on the plastic properties of corticostriatal connections as the core processing units for basal ganglia activity. In this scenario, growing evidence suggests the involvement of glial cells in synaptic transmission, synaptic plasticity, and synaptic remodeling, both in the post-natal and adult brain (103–106). Glial cells, including astrocytes, microglia, oligodendrocytes, and other specialized cells, appear as a highly represented cellular population throughout the CNS (107–109). The functional architecture of the neuronal-glial network has been deeply investigated during the last years in different brain structures, including basal ganglia (110, 111). Glia/neuron ratio was found to vary in the human brain in relation to neuronal density and the numerical relationship between these cellular elements was found to be remarkably conserved among different species, as if a proper balance is essential for the physiological brain activity (107, 112). It has been shown that the overall ratio between non-neuronal/neuronal cells in the whole human brain is close to 1, varying from a value of 1.48 in the gray matter of the cerebral cortex to 11.35 in basal ganglia/diencephalon/mesencephalon/pons (113). Accordingly, an updated view of the striatal network function should necessarily take into account the contribution of glial cells during both physiological and pathological conditions.

## Neuron-Astrocyte Interactions in the Striatum

Astrocytes are widely represented in the brain, counting approximately 19-40% of total brain cells (112) and exerting multiple homeostatic functions through thousands of fine processes, creating “bushy” territories around neuronal somata, dendrites, and blood vessels (110). The functional view of these cells has significantly changed after the discovery that astrocytes can display a form of cellular excitability based on variations of intracellular calcium ion (Ca^2+^) concentration (114, 115), occurring spontaneously and in response to neurotransmitter release by neighboring synaptic connections (116, 117). The identification of a bidirectional neuron-astrocyte communication led to identify synaptic connections as “tripartite” elements, where astrocytes represent cellular processors of information with selective responses to specific synaptic inputs and integrative abilities due to cell-intrinsic properties and nonlinear input-output relationships (104). A recent study has identified, through cortical live-cell 3D-STED microscopy in mice, astrocytic Ca^2+^ signals at the level of bulbous enlargements localized along the thin astrocytic processes (118). Such “nodes” have been found to be in tight contact with dendritic spines, suggesting the presence of specific signaling domains tailored for neuron-astrocyte communication (118). The astrocytic processes can contact neighboring synapses and create an “astroglial cradle” essential for synaptic maturation and isolation (119), influencing neuron transmission through different mechanisms, including vesicular gliotransmission, release of neuroactive substances, potassium buffering, and neurotransmitter recycling (103, 120–122).

It has been shown that astrocytes may influence synapse structure and function through several contact-mediated and soluble synaptogenic cues (123). Specifically, astrocytes may regulate cortical synaptogenesis through the secretion of thrombospondins (TSP1 and 2) (124) or through cell adhesion proteins like gamma protocadherins (125). Other astrocyte-derived soluble mediators may modulate the expression of neurotransmitter receptors at synaptic sites since heparan sulfate proteoglycans glypican 4 and 6 (Gpc4 and 6) were linked to an increased expression of GluA1 AMPA receptor (AMPAR) subunit at the post-synaptic level (126) and tumor necrosis factor α (TNF-α) was associated with enhanced surface expression of AMPARs in hippocampal neurons (127).

Interestingly, CNS astrocytes are not a homogeneous cell population, displaying different region-specific functions to optimize local neural network activity (110, 128, 129). Transcriptomic and proteomic analysis revealed significant differences in gene expression patterns between striatal and hippocampal astrocytes (111). Moreover, from a morphological point of view, it has been shown in murine tissues that striatal and hippocampal astrocytes are characterized by equivalent somatic volumes, number of primary branches, and cell volumes, but striatal astrocytes displayed larger territory volumes impinging upon greater numbers of neurons (129). In line with these findings, other authors have recently found, through a genetically targeted neuron-astrocyte proximity assay (NAPA), that murine striatal astrocytes tightly interact with cortical, thalamic, and nigral projections (130). Interestingly, no substantial anatomical difference was found in astrocyte-synapse proximity for D1- and D2-like receptor-expressing MSNs (130). Overall, it has been estimated that each striatal astrocyte could make contact with an average number of ~11 MSNs, sampling D1- and D2-like receptor-expressing MSNs in an almost equivalent way (130), and could interact with approximately 50,700 excitatory synapses (129).

This extensive net of neuron-astrocyte interactions could exert a key role in the regulation of striatal network function. Indeed, it has been hypothesized that homogeneously distributed striatal astrocytes could display different patterns of activation in order to sustain and modulate the coordinated activity of direct and indirect striatal pathways (131). It has been shown that homotypic (D1-D1 or D2-D2) but not heterotypic MSNs stimulation is characterized by an endocannabinoid-dependent activation of astrocytic CB1 receptors (CB1Rs), leading to glutamate release upon elevating their Ca^2+^ levels (131). Of note, glutamate released by corticostriatal projections and by astrocytes could directly stimulate glutamatergic NMDA receptors (NMDARs) expressed at the synaptic cleft by MSNs (131), but it could also act on metabotropic receptor subtype 5 (mGluR5) expressed by astrocytes (132). The activation of these astrocytic receptors could lead to an additional Ca^2+^-dependent release of glutamate, triggering a stimulation of MSNs through GluN2B containing NMDARs which could last for minutes beyond the initial stimulus (133). In this scenario, the selective reinforcement of homotypic synapses supports the presence of specific astrocyte subpopulations enhancing striatal pathways divergence and coordination during behavior/action execution (131).

Astrocytes can also influence the induction of corticostriatal synaptic plastic changes, such as LTD (134) or STDP (99), through neurotransmitter release or regulation of glutamate reuptake. Astrocytes ensure a proper striatal signal-to-noise ratio, regulating glutamate concentration in the synaptic cleft (135), and this activity could act as a gatekeeper for the induction of corticostriatal synaptic plasticity. It has been hypothesized that the proper activation of astrocytic excitatory amino-acid transporter type-2/glutamate transporter 1 (EAAT2/GLT-1) could represent a key element for ensuring the detection of the temporal contingency required for Hebbian synaptic plastic changes like STDP, since the blockade or the overexpression of this astrocytic protein could lead to aberrant synaptic plastic changes (99).

Moreover, other authors proposed a model in which up-state MSNs could stimulate neighboring astrocytes through dendritic GABA release, leading to a GABA_B_ receptor- and Gi-dependent release of Ca^2+^ from intracellular stores (136). The activation of this transduction pathway is supposed to upregulate the synaptogenic cue thrombospondin-1 (TSP1) in astrocytes, boosting excitatory synapse formation, fast excitatory synaptic transmission and MSNs firing frequency in the striatum (136). The abnormal activation of this Gi-dependent astrocytic pathway could pathologically enhance corticostriatal transmission leading to behavioral hyperactivity and impaired attention in mice (136). In line with these observations, an alteration of astrocytic Ca^2+^ dynamics has been linked with abnormal MSNs activity and excessive self-grooming behavior, as assessed with *in vivo* electrophysiological recordings in mice (137).

Overall, the astrocytic modulation of STDP through reinforcement signals and the maintenance of a proper signal-to-noise ratio, allowing the detection of the temporal relationship between two paired stimuli (negative or positive pairing), could deeply influence the basal ganglia action-outcome synaptic associations. Accordingly, altered astrocytic activity has been linked to enhanced reward-seeking behavior and to the pathological intake of drugs of abuse (138). In rodent models, methamphetamine and cocaine assumption has been associated with a reduction in the contacts between astrocytes and synapses in the NAc (139, 140), while cocaine and heroin seem to be linked to reduced expression of the glutamate transporter EAAT2/GLT-1, mainly located in astrocytes (141, 142). A recent study showed that DA in the NAc could directly stimulate astrocytes through D1-like receptors (D1Rs), with a subsequent astrocytic release of ATP/adenosine and inhibition of excitatory transmission through the stimulation of pre-synaptic A1 receptors expressed by glutamatergic projections (143). This evidence suggests new possible astrocyte- and dopamine-dependent pathways regulating reward-related behaviors.

Lastly, the neuron-astrocyte net could represent an anatomical “track” facilitating microglial cells movements and synaptic surveillance (130). Indeed, astrocytes may participate in network modeling and synapse elimination *via* direct synaptic phagocytosis and in cooperation with microglial cells, tagging synapses for elimination through the secretion of transforming growth factor-β (TGF-β) (144, 145).

## Microglial Cells as Modulators of Striatal Synaptic Function

Microglia can account for 5%–12% of total cellular elements in the CNS, representing the main element of the resident CNS immune system with critical roles in organizing rapid responses against different kinds of tissue injury (146–148). Activated microglial cells can produce soluble chemotactic and pro-inflammatory molecules orchestrating inflammatory responses within the CNS, and these cells can assume a phagocytic profile aimed at clearing cellular debris (146–148). During the last decades, it has been clearly established that microglial cells are physiologically involved in synaptic transmission, plasticity and structural remodeling during CNS development and adult life, dynamically interacting with synapses as “synaptic sensors” (105, 149, 150).

Several high-resolution imaging studies have shown that immature, redundant, or dysfunctional axonal terminals and dendritic spines can be engulfed by microglial cells as a mechanism to refine brain networks (149, 151). Such a dynamic process of synaptic pruning was found to be dependent on several potential ‘find-me’ and ‘eat-me’ neuro-immune pathways (152). The chemokine CX_3_CL1, which could be secreted or expressed as a membrane-tethered protein, is thought to represent a synaptic tagging mechanism through which neuronal cells may attract resident CX_3_CR1-expressing microglia (153, 154). Accordingly, the genetic ablation of CX_3_CL1 was associated with an increased density of immature synapses in cortical areas (151, 155) and impaired cortical synaptic remodeling (154). Another potential ‘eat-me’ signal may be represented by the classical complement proteins C1q and C3, which can be expressed in an activity-dependent manner by neuronal cells in less active or immature synapses, flagging them for removal by microglia (149, 152, 156). Of note, the expression of complement proteins at synaptic sites is influenced by astrocyte-derived TGF-β (144), suggesting a cooperation between these glial elements in the process of synaptic pruning. Such active neural network refinement is thought to be involved in learning and memory processes, mediating the removal of specific synaptic connections as a way of active forgetting (157–159). The microglia- and complement-dependent process of synaptic shaping could be crucial in maintaining a physiological balance between retaining relevant memory engrams and removing irrelevant ones (160), and it could be pathologically enhanced in various CNS diseases characterized by learning/memory deficits, including Alzheimer’s disease (158, 159).

In addition, beyond synaptic removal, microglial cells may participate in the functional modulation of synaptic transmission and plasticity by producing soluble immune mediators, including the pro-inflammatory cytokine interleukin-1β (IL-1β) (161) or neurotrophic factors like brain-derived neurotrophic factor (BDNF) (162). Moreover, inflammatory processes of the CNS may be accompanied by a microglia-dependent disruption of neuronal plastic properties, relying on the microglial over-expression of reactive oxygen species (ROS)-producing enzymes such as NADPH oxidase (163). In this scenario, the molecular mechanisms underlying microglia-centered neuro-immune pathways have been mostly investigated in cortical and hippocampal areas, with few reports regarding its involvement in the striatal network (152).

Murine microglial cells display a spectrum of distinct anatomical features, lysosome content and membrane properties across basal ganglia nuclei, suggesting that region-specific local cues could shape the functional state of these cells (164). A recent study described a role for microglia and complement in sex-specific synaptic shaping in the NAc during rat adolescence, with potential key consequences on social behavior (165). Specifically, in adolescent male rats, D1Rs in the NAc were found to be downregulated and degraded through a microglial and C3R-dependent engulfment of C3-tagged D1Rs. Interestingly, the reduced expression of this receptor at synaptic sites in the NAc was correlated with social play behavior in male rats, and the pharmacological interference of C3–C3R interactions was able to increase social play in a D1R-dependent manner (165). This evidence raises the hypothesis that an immune-mediated shaping of striatal synapses may regulate behavioral responses in an age-related way (165).

Moreover, microglial cells could modulate goal-directed and drug-seeking behavior through a molecular pathway, yet to be fully characterized, involving a Toll-like receptor 4 (TLR4)-induced modulation of NMDAR-dependent synaptic plasticity in MSNs of NAc (166), or through the secretion of soluble inflammatory mediators exerting neuromodulatory effects on excitatory striatal transmission, such as TNF-α (167). Interestingly, it has been shown that microglial cells express dopaminergic receptors and DA can modulate the activation of these cells (168–170). Indeed, the release of TNF-α can be induced by the activation of microglial DA D2 receptors (D2Rs) (167). A proper investigation of the molecular pathways linking microglia and striatal synaptic transmission will help understand the puzzling neuro-glial interactions within the basal ganglia network.

## Soluble Immune Molecules as Striatal Neuromodulators

Increasing evidence suggests that soluble products of inflammation can influence learning/memory processes and human behavior through the modulation of synaptic transmission and plasticity in different neural networks (2, 3, 5, 6, 171, 172). In this context, the effects exerted by pro-inflammatory cytokines have been mainly investigated in hippocampal and cortical areas, suggesting that some of these molecules could play a key physiological role in memory formation, storage and retrieval (3, 172). The connection between IL-1β production and hippocampal synaptic plasticity induction can be considered as paradigmatic in the field. Synaptic LTP in hippocampal areas was found to be followed by IL-1β gene expression and the genetic ablation or the pharmacological blockade of the IL-1β axis were found to alter the induction and maintenance of hippocampal synaptic long-term changes, together with the execution of hippocampal-dependent memory tasks (173–176). Interestingly, IL-1β-dependent modulation of synaptic plasticity seems to rely on several mechanisms, including the modulation of NMDARs and AMPARs phosphorylation, synaptic localization, and calcium conductance (177–180). Similarly, other cytokines like interleukin-6 (IL-6) (181–184), interleukin-18 (IL-18) (185–187), interferon-γ (IFN-γ) (188, 189), TNF-α and -β (127, 190–196) and, more recently, interleukin-17 (IL-17) (197–199) have been described as key cortical neuromodulators influencing synaptic transmission and plasticity during physiological and pathological conditions, with relevant behavioral and cognitive implications.

On the other side, less is known about the potential effects of such molecules on subcortical network activity. It has been suggested that IL-1β could be involved in the modulation of striatal neurotransmission during both physiological and pathological conditions. Indeed, the exposure of murine corticostriatal slices to IL-1β is followed by an enhanced frequency of spontaneous excitatory transmission, an effect potentially caused by the activation of transient receptor potential vanilloid 1 (TRPV1) channels located in striatal pre-synaptic terminals (200) together with a reduced sensitivity of CB1Rs controlling glutamate release (201). Interestingly, exposure to IL-1β could induce an hyperactivation of MSNs not only through the enhancement of glutamatergic neurotransmission but also by lowering GABAergic inhibition of MSNs (202). In this case too, the modulation of pre-synaptic TRPV1 channels and CB1Rs seems to mediate the IL-1β-dependent modulation of inhibitory terminals (203, 204). The disruption of striatal glutamatergic/GABAergic balance through the IL1β-CB1R axis could play a key role in triggering altered motivated behavior during pathological neuroinflammation, with a possible additional effect linked to an IL-1β-dependent alteration of dopaminergic transmission or DA-induced release of neurotrophic factors (205, 206). Of note, nigral dopaminergic neurons were found to express IL-1 receptors (207), thus suggesting a potential direct modulation of striatal dopaminergic projections.

Another pro-inflammatory cytokine, TNF-α, has been hypothesized to modulate glutamatergic neurotransmission in the striatum. Specifically, exposure of murine brain slices to TNF-α was able to induce an alteration of spontaneous excitatory synaptic currents in MSNs, with an increased duration and decay time that could be reversed by the application of anti-TNF receptor (TNFR)–antibodies (208). Interestingly, the same study has shown that pathological neuroinflammation in mice is characterized by similar striatal synaptic changes in association with an abnormal microglial release of this cytokine and an increased expression and phosphorylation of AMPARs in MSNs (208). These results suggest a potential TNF-α-dependent modulation of glutamatergic neurotransmission, in line with what has been described in other brain areas (209–211). In support of this hypothesis, intracerebroventricular (icv) injections of TNF-α were able to enhance striatal glutamatergic transmission, mimicking the synaptic alterations observed during pathological neuroinflammation (212). Of note, such modifications in striatal excitatory neurotransmission were paralleled by behavioral abnormalities that could be reversed by the icv administration of anti-TNF-α drugs (212).

On the other hand, it has been proposed that TNF-α could play a role in the homeostatic maintenance of excitatory synaptic weights around a firing set point, exerting a physiological and adaptive role aimed at limiting the corticostriatal drive during pathological conditions (213). Specifically, Lewitus and coworkers have shown that TNF-α is upregulated after the prolonged disinhibition of MSNs through the blockade of D2Rs, and can drive AMPARs internalization, DARPP-32 and GluA1 dephosphorylation in these cells (213) collectively reducing corticostriatal synaptic input as an adaptive homeostatic response. Taking into account that this cytokine was found to induce an opposite and rapid exocytosis of AMPARs in hippocampal, motor and visual cortex neurons (214–217), it could be hypothesized that TNF-α could exert region- and neuronal type-specific modulations of activity-dependent synaptic upscaling or downscaling (218). Moreover, considering that the stimulation of microglial D2Rs was found to induce the production of TNF-α by these cells (167), this TNF-α-centered neuro-immune interaction could be involved in the well-known D2R-dependent inhibition of corticostriatal transmission. An abnormal striatal release of this cytokine during neurodegenerative and neuroinflammatory disorders could impair the physiological tuning of corticostriatal inputs, altering basal ganglia activity with potential cognitive and behavioral abnormalities.

Lastly, interferons (IFNs) represent a family of soluble immune mediators exerting pleiotropic immune-modulating effects, with particular regard to immune-surveillance processes against viral infections (219, 220). Interestingly, IFNs can be produced by different cellular subtypes in the CNS and can directly modulate neuronal function and synaptic transmission leading to cognitive and psychiatric disturbances during infectious or inflammatory disorders of the CNS (189, 221–223). Several drugs have been designed to mimic the immunomodulating effects of IFNs, in order to treat human disorders characterized by a pathological immune system activation (219). In this scenario, it has been shown that IFN-β1a is able to reduce the amplitude of excitatory synaptic currents in MSNs, suggesting an inhibitory effect on glutamatergic transmission in the striatum (224). In particular, this cytokine was found to specifically influence NMDAR-mediated synaptic currents in MSNs, interacting with the GluN2A subunit of this receptor, with no effect on the AMPAR-dependent component of striatal excitatory transmission (224). Interestingly, it has been hypothesized that the effect of IFN-β1a on synaptic transmission relies on the activation of post-synaptic Ca^2+^/Calmodulin(CaM)-dependent protein kinase II (CaMKII) (224), known to strictly interact with NMDAR and GluN2A subunit (225, 226). Of note, the exposure of murine brain slices to IFN-β1a was found to reduce the detrimental consequences induced by mitochondrial complex I inhibition in the striatum, through the modulation of the IFN-activated intracellular JAK-STAT1 pathway (227).

## A Synaptocentric Perspective: Striatal Neuroinflammation and Neurological Disorders

The last decades have been characterized by an intense investigation of the crucial role played by neuroinflammation in the pathogenesis of several neurodegenerative disorders (228, 229), including the prototypical disorder of the basal ganglia network: Parkinson’s disease (PD) (230). Mounting evidence suggests that an aberrant immune system activation and a chronic inflammatory process within the CNS may contribute to the progressive loss of midbrain dopaminergic neurons characterizing PD (231–234). Pathological studies showed that midbrain infiltrating T cells (235) and increased basal ganglia levels of proinflammatory cytokines, such as TNF-α, IL-1β and IL-6 (236, 237), can be found in post-mortem brains of PD patients. Moreover, several studies have reported increased levels of pro-inflammatory cytokines in serum and cerebrospinal fluid (CSF) of PD patients (238–242). In this scenario, brain infiltrating immune cells could represent autoreactive T lymphocytes targeting alpha-synuclein (α-syn) aggregates, as suggested by a recent study (243), or different and still unknown neuronal antigens, orchestrating a pathological inflammatory reaction through the production of chemo-active and pro-inflammatory molecules. Several studies performed in experimental PD models have led to hypothesize that such abnormal immune activation in the basal ganglia and the midbrain could act as a co-factor in PD-associated neurodegeneration, by triggering cell-to-cell death signals or because of the toxic damage induced by soluble pro-inflammatory cytokines (235, 244–250).

Resident immune cells are thought to be involved in these pathological processes. Early reports showed high levels of activated microglia in the midbrain and in the striatum of PD brains (251, 252), and the temporal relationship between the presence of inflammation with activated microglia and the emergence of α-syn pathology has been recently investigated in dopaminergic neuronal grafts implanted in the striatum of PD patients (253). Interestingly, the authors have found evidence of inflammation long before the accumulation of α-syn, supporting the concept that microglia plays an integral role in the propagation and spread of α-syn pathology (253). Studies performed in experimental models supported the hypothesis of a microglial-driven degeneration of dopaminergic neurons during the disease, probably due to an increased production of reactive oxygen species and soluble mediators like TNF-α and IL-1β (254–260). Similar neurotoxic effects have been described for chemokines and cytokines released by the reactive and dysfunctional astrocytes that have been identified in human and experimental PD (261, 262) since the early phases of the disease (263). However, most of the studies have focused on the pathological neuro-immune interactions potentially triggering progressive dopaminergic neuronal death during PD, overlooking the possible detrimental influence exerted by activated immune cells on synaptic connections within the basal ganglia network, which could also anticipate irreversible cell loss.

**Figure 2:**
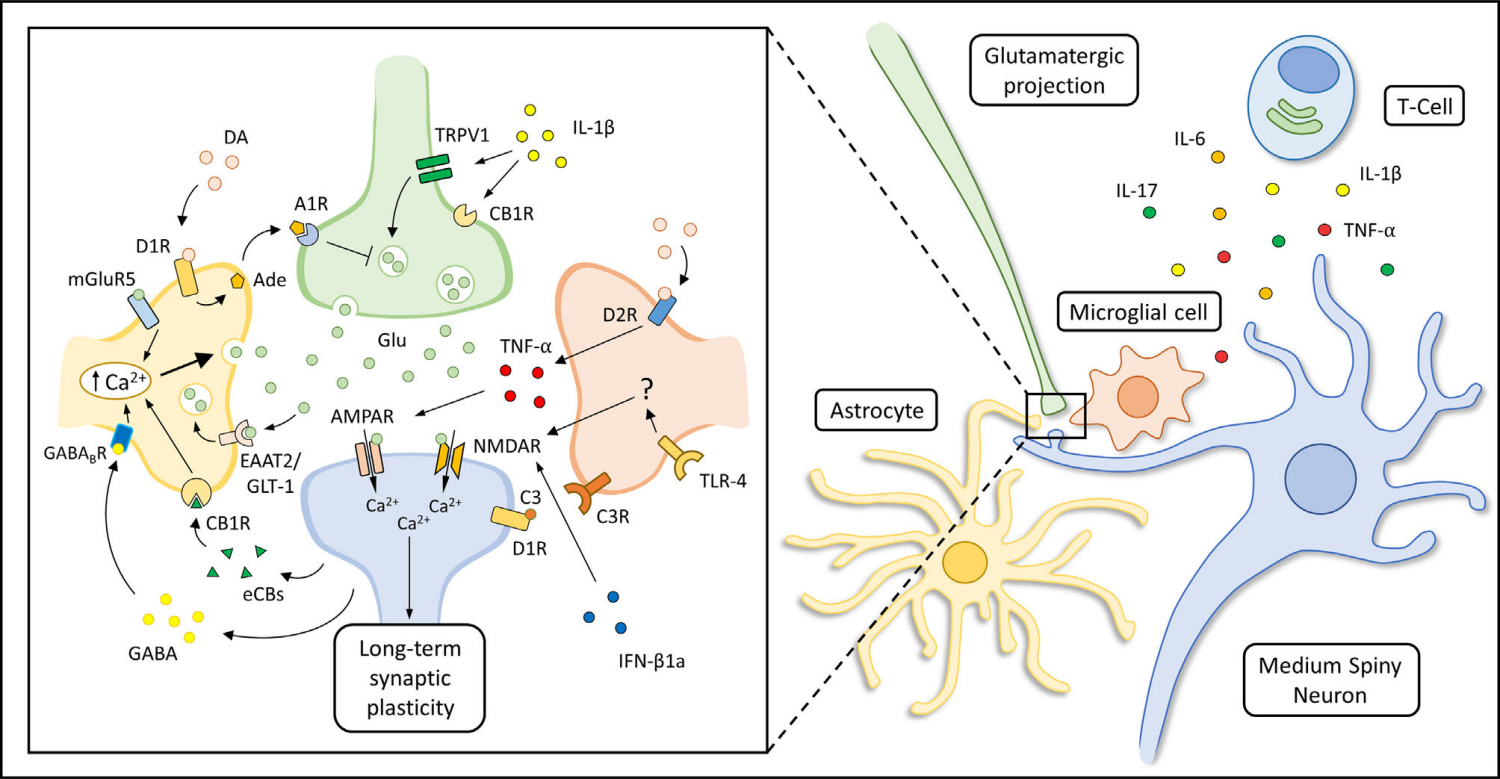
Immune modulation of striatal synaptic transmission. Suggested mechanisms underlying astrocytic, microglial and immune modulation of corticostriatal synaptic transmission are represented in the box on the left. The production of soluble immune mediators (like IL-1β, TNF-α, IL-6 and IL-17) by activated T-cells, astrocytes and microglial cells can influence striatal transmission during the course of neuro-psychiatric disorders. Specifically, IL-1β can enhance striatal excitatory transmission activating transient receptor potential vanilloid 1 (TRPV1) channels (200) and reducing CB1 receptors (CB1Rs) activation (201) at pre-synaptic glutamatergic terminals. In addition, it has been shown that TNF-α can increase the decay time and duration of spontaneous striatal excitatory transmission during pathological neuroinflammation (208) or induce AMPAR internalization as an adaptive response to prolonged MSNs disinhibition (167, 213). Microglial cells can also regulate dopamine D1R expression through a complement (C3-C3R) dependent internalization and degradation of this receptor (165). Other authors have shown that IFN-β1a can inhibit NMDAR-mediated glutamatergic transmission interacting with NMDAR subunit and CaMKII (224). Glutamate (Glu) released in the synaptic cleft could activate AMPARs and NMDARs of MSNs, but could also act on metabotropic receptor subtype 5 (mGluR5) expressed by astrocytes triggering a Ca^2+^-dependent release of Glu, sustaining MSNs activation for minutes after the initial stimulus (133). Depolarization of MSNs is associated with endocannabinoids (eCBS) release which can activate astrocytic CB1Rs leading to an increase of intracellular Ca^2+^ levels and glutamate (Glu) release (131). Moreover, up-state MSNs could lead to Gi-coupled GABA_B_ receptor activation in neighboring astrocytes through dendritic GABA release (Nagai et al., 2019). The activation of this astrocytic receptor is thought to induce astrocytic Ca^2+^ release from cellular stores and influence striatal excitatory transmission through the production of the synaptogenic cue TSP1 (not shown in the figure). The astrocytic expression of EAAT2/GLT-1 is thought to be required for the maintenance of a proper Glu concentration in the synaptic cleft. Astrocytic Glu reuptake allows the detection of the temporal contingency of synaptic stimuli, modulating the induction of corticostriatal synaptic plasticity (99). In addition, dopamine (DA) could trigger the release of ATP/adenosine (Ade) by astrocytes through D1Rs activation, leading to A1 receptor (A1R)-dependent inhibition of striatal excitatory transmission (143). Microglial cells can modulate NMDAR-dependent synaptic plasticity in MSNs through a still not fully characterized Toll-like receptor 4 (TLR4)-dependent mechanism (166), or influence glutamatergic transmission through the secretion of tumor necrosis factor α (TNF-α), which could be also induced by the activation of microglial D2Rs (167).

As introduced above, the abnormal release of pro-inflammatory soluble mediators within the striatum, together with the loss of the physiological supporting functions exerted by glial cells, could alter the function of the striatal synaptic network during PD development, long before the occurrence of neurodegenerative features (**Figure 2**). Accordingly, it has been hypothesized that a striatal immune-mediated synaptopathy could account for disabling cognitive, motor and behavioral abnormalities in PD patients (264), which can be highlighted in disease stages characterized by a still partial dopaminergic cell loss (265). In this scenario, several studies performed in experimental models of PD have highlighted that synaptic dysfunction can be considered as an early event in the pathogenesis of the disease, altering the ability of corticostriatal connections to express short- and long-term plastic changes (266–268). The disruption of the physiological filtering activity of the basal ganglia network could induce an extensive reorganization of the overall architecture of brain node connectivity since early disease stages, as shown by functional imaging studies (269, 270).

To date, PD-related synaptopathy has been mainly linked to the synaptic and molecular effects of pathological α-syn aggregates (271–278). However, an involvement of the immune system in the synaptic dysfunction triggered by α-syn accumulation cannot be ruled out since α-syn can activate different subsets of T-cells (243) and oligomeric or fibrillary α-syn can induce a pro-inflammatory activation of microglia through the interaction with toll-like receptor 2 (TLR2) (279) or the activation of NF-κB pathway (262). Moreover, it has been suggested that astrocytes can uptake and accumulate the pathological α-syn released by neighboring neuronal cells (280), triggering a pro-inflammatory astrocytic reaction with the production of soluble molecules such as IL-1β, IL-6 and TNF-α (281). Collectively, accumulating evidence suggests that striatal neurons and synapses could be submerged in an inflammatory micro-environment linked to α-syn aggregation (282), with the exposure to soluble immune molecules with demonstrated modulatory effects on corticostriatal terminals. Such abnormal immune influence on striatal transmission could also account for late disease complications, like L-DOPA-induced dyskinesia (283), which pathogenesis is thought to rely on an abnormal corticostriatal synaptic plasticity (284–286).

The potential relevance of pathological neuro-immune interactions during PD is supported by a recent study showing that corticostriatal synaptic plasticity can be rescued through the modulation of astrocytic and microglial activation by transcranial magnetic stimulation (TMS) (287). Specifically, it has been shown that the loss of LTD and LTP of corticostriatal projections accompanying striatal dopaminergic denervation can be restored by TMS treatment in an experimental model of PD (287). The beneficial effects of TMS on synaptic function were paralleled by an increase in striatal DA levels and an amelioration of PD-related deficits in motor behavior. Interestingly, such a therapeutic protocol was also associated with a significant reduction of astrocytic and microglial pro-inflammatory responses in the striatum (287). This result is of particular relevance since glial cells have been proposed as key targets and effectors of TMS protocols, potentially mediating widespread effects in neural networks through extensive connections and cell type-specific modulation of neuronal firing (288). The TMS-dependent reduction of pathological glial activation in the striatum could lower the production of soluble pro-inflammatory mediators and lead to the recovery of glial supporting functions relevant for neurons, like the modulation of DA and glutamate metabolism, reuptake, or release (138, 289). In line with these findings, another research group has shown that the modulation of astrocytic glutamate content and reuptake in the globus pallidus pars externa (GPe) is able to restore the proper pre-synaptic tuning of striato-pallidal input in an experimental model of PD (290). This result is interesting since the hyperactivity of striato-pallidal pathway is thought to underlie hypokinetic features of PD patients (291, 292) and the astrocytic gating of these synapses, which is disrupted during PD, can represent an alternative therapeutic strategy (290).

Further investigations are required to clearly decipher the potential dysfunction of striatal astrocytes during the course of PD, but findings obtained in research studies on other basal ganglia disorders like HD seem to suggest that the loss of physiological astrocytic properties could be associated with altered action selection, habit formation, impulse inhibition and motor behavior (111, 293). HD is a genetic disorder primarily affecting cortico-basal ganglia-thalamo-cortical network, linked to a pathological expansion of a polyglutamine-encoding CAG repeat in the huntingtin gene (HTT) and characterized by progressive motor hyperactivity with psychiatric and cognitive disturbances (294, 295). Accumulating evidence, both clinical and pre-clinical, suggests that an alteration in astrocytic activity could be deeply involved in the pathogenesis of HD (261, 293). In post-mortem tissues obtained from HD patients, the striatum was characterized by a significant astrocytic reaction (296) with an altered expression of the transporter EAAT2/GLT-1, potentially triggered by mutant HTT (mHTT) (297–299). Interestingly, the delivery of mHTT-expressing human glial cells to the striatum was able to cause an HD-like phenotype in mice, with the evidence of MSNs hyperexcitability and striatum-dependent motor impairment (300). Conversely, the selective deletion of mHTT in astrocytes was found to be protective in an experimental model of HD, with beneficial effects on motor and psychiatric-like disturbances (301). Overall, it has been hypothesized that an altered expression of astrocytic proteins, such as Kir4.1 and EAAT2/GLT-1, together with an impairment in astrocytic Ca^2+^ signaling, could alter MSNs membrane excitability and disrupt cortico-striatal glutamatergic transmission (111, 293). Such disease-related synaptic and neuronal abnormalities could influence the physiological mechanisms underlying basal ganglia’s ability to inhibit context-inappropriate actions, leading to the typical excessive and uncontrolled motor behavior of HD patients. In line with this hypothesis, the pathological activation of striatal glial cells has been proposed as a key pathogenic factor in neuropsychiatric disorders characterized by repetitive and impulsive behaviors like OCD, TS and “pediatric autoimmune neuropsychiatric disorders associated with streptococcal infections” (PANDAS) (302, 303), potentially inducing a disruption of synaptic tuning in the striatal network. In this scenario, the neuronal and synaptic consequences triggered by the acquisition of a pro-inflammatory phenotype by striatal astrocytes and the role of microglial cells during HD are still under investigation (261, 304). Overall, interventions aimed at lowering the immune system activation and restoring the physiological glial functions could counteract the synaptic imbalance characterizing basal ganglia disorders, limiting early clinical features, late disease complications and, potentially, disease progression in several neurological and psychiatric diseases.

## Concluding Remarks

The functional view of the basal ganglia network slowly moved from the brain motor control station to the decision-maker of appropriate context-dependent cognitive, behavioral and emotional responses. In parallel, the physiological processes underlying the integrative and filtering activity of the basal ganglia started to be deciphered during the last decades. Plastic properties of striatal synaptic connections have been demonstrated as crucial for the integration of multimodal cortical inputs and for conveying a proper basal ganglia output driving an individual’s action selection/inhibition and habit formation. In this scenario, the current evidence on the neuro-modulatory role played by immuno-glial cells in cortical areas suggests that corticostriatal projections and subcortical networks can be influenced by the immune system as well.

The characterization of the neuro-immune interactions taking place in the striatum, both in its dorsal and ventral areas, could help to decipher the molecular mechanisms underlying the previously underscored effects of the immune system on motivated and context-dependent human behavior. The identification of cells and soluble immune mediators involved in the striatal neuro-immune cross-talk could lead to a new approach to basal ganglia disorders, disclosing a novel pathophysiological view for motor, behavioral, cognitive and emotional abnormalities accompanying neurological and psychiatric disorders.

## Author Contributions

MDF conceived the review. AM performed the literature review, wrote the manuscript draft and prepared the figures. MDF, VG, LP, and PC reviewed and integrated the manuscript draft and the figures. All authors contributed to the article and approved the submitted version.

## Funding

This work has been supported by the Marlene and Paolo Fresco Institute for Parkinson’s and Movement Disorder, Fresco Parkinson Institute, New York University School of Medicine.

## Conflict of Interest

AM received travel grants from Biogen, Novartis, Merck, Teva, Almirall and Sanofi to attend national and international conferences. MDF participated on advisory boards for and received speaker or writing honoraria and funding for traveling from Bayer, Biogen Idec, Sanofi, Merck, Mylan, Novartis, Roche and Teva. PC participated on advisory boards for and received funding for traveling, speaker honoraria and research support from AbbVie, Biogen Idec, Merck, Genzyme, Novartis, Prexton, Teva, UCB and Zambon.

The remaining authors declare that the research was conducted in the absence of any commercial or financial relationships that could be construed as a potential conflict of interest.
